# Comparision of nerve stimulator and ultrasonography as the techniques applied for brachial plexus anesthesia

**DOI:** 10.1186/1755-7682-4-4

**Published:** 2011-01-21

**Authors:** Beyazit Zencirci

**Affiliations:** 1 Kahramanmaras Sutcu Imam University Medical Faculty, Department of Anesthesiology and Reanimation Kahramanmaras, Turkey

## Abstract

**Background:**

Brachial plexus block is useful for upper extremity surgery, and many techniques are available. The aim of our study was to compare the efficacy of axillary brachial plexus block using an ultrasound technique to the peripheral nerve stimulation technique.

**Methods:**

60 patients scheduled for surgery of the forearm or hand were randomly allocated into two groups (n = 30 per group). For Group 1; US, and for Group 2 PNS was applied. The quality and the onset of the sensorial and motor blockade were assessed. The sensorial blockade, motor blockade time and quality of blockade were compared among the cases.

**Results:**

The time needed to perform the axillary brachial plexus block averaged is similar in both groups (p > 0.05). Although not significant statistically, it was observed that the sensory block had formed earlier in Group 1 (p > 0.05). But the degree of motor blockade was intenser in Group 1 than in Group 2 (p < 0.05).

**Conclusions:**

Ultrasound offers a new possibility for identifiying the nerves of the brachial plexus for regional anesthesia. The ultrasound-guided axillary brachial plexus block is a safe method with faster onset time and better quality of motor blockade compared to peripheral nerve stimulation technique.

## Introduction

Regional anaesthesia can be defined as removing nerve conduction and pain at the certain parts of a body without causing sensory loss [1]. A method of regional anaesthesia used for this purpose is brachial plexus block that is applied in operations to be carried out on the 1/3 distal part of upper extremities as well as hands, forearms and arms. It is known that the first brachial plexus block was applied in 1884 by RJ Hall upon exploration and sight of the plexus and by injecting cocaine to provide infiltration anaesthesia. Many other methods have been described until today since Hirschel's application of the blinding axillary block in 1911 [2,3].

Brachial plexus can be blocked through various anatomical approaches such as interscalene, supraclavicular, infraclavicular and axillary approaches. Axillary block techniques can be applied by using transarterial fixation, paresthesia or nerve stimulator [4]. Current techniques available for nerve localization mark anatomical indicators for the estimated location of brachial plexus. As well as causing anxiety in the patient and long application processes, blinding techinques may also cause nerve damages, vein perforations and complications such as systemical local anaesthetic toxic reactions. Nerve stimulator technique, however, ensures that the needle is correctly placed without causing paraesthesia. Ultrasonography allows us to display brachial plexus with a higher quality and helps nerve localization and these can increase the quality of the nerve block. Through ultrasonography, peripheric nerves, needle localization and local anaesthetic distribution, that is required for a successful conduction block, can be directly displayed [5].

In our study, we have aimed to compare the sensory and motor block effects of peripheral nerve stimulation (PNS), that facilitates the application of axillary brachial plexus block (AXB) and increases the prospects, and the technique of ultrasonography (US) that has recently been put into use.

## Methods

Having obtained the required written consents both from the Ethics Committee and 60 patients consisting the ASA I-II groups, we decided to divide this 60 patients, who were planned to go under extremity operations through the application of AXB, into two equal groups of 30 cases. Those with history or presence of cardiac, inspiratory and/or renal failures and those who are pregnant were not included in the study. No premedications were applied to the cases.

An intravenous cannula was inserted into the contralateral arm, and a continuous infusion (crystalloid solution) was started. For the whole procedure the patients were routinely monitored with electrocardiogram (ECG), non-invasive blood pressure (NIBP) measurement, and pulse oximetry (SpO_2_).

AXB was carried out by abducting the arm, on which the block will be applied, in way to create a 90°angle with the body and by flexing and externally rotating the forearm so that the hand can be placed right next to the head and the palm can be positioned as facedown. Following the positioning of the cases in both groups, the area on the axillary region to be operated was disinfected.

After the appropriate positioning of the Group-1-cases and following the completion of the required preparations, a 22 G insulated needle (Stimuplex^® ^D 50 mm, B.Braun, Germany) was inserted into the axillary region under sterile conditions and in company with ultrasonography (by using Aloka^® ^SSD-4000, Japan, 10 Mhz prob). First radial, next median, thirdly ulnar and lastly musculocutaneous nerves were identified. After identification of each nerve, 7-10 ml LA (In total 40 ml of 0.75% Ropivacaine for the four nerves) was injected until the nerve was completely surrounded.

As for the cases of Group 2, following the appropriate positioning and completion of the required preparations, similar to the other group, in total 40 ml - for each nerve 7-10 ml - of 0.75% Ropivacaine was injected by using nerve-stimulator-specific, sterile, teflon-isolated needles (22G insulated needle) (Stimuplex^® ^D 50 mm [15°]) in company with the available nerve stimulator (Stimuplex^® ^Dig RC, B.Braun, Melsungen, Germany) and at the same time, the motor response given by the nerves that form the brachial plexus to nerve stimulation was also considered (radial: *arm and finger extension, supination; *median: *wrist, 2nd and 3rd finger flexion, pronation*; unlar: *4th and 5th finger flexion, thumb adduction*, musculocutaneous: *arm flexion*).

After the end of the AXB, the anesthetist performing the block evaluated sensory and motor block as follows: every five minutes and for 30 minutes the innervated areas (each dermatome) was evaluated using a pinprick. When the needles were no longer felt, cutaneous anesthesia was considered to be present. The motor block was evaluated once at the end of the 30 minute period. The motor block was estimated as being 0, 33, 66 or 100%: 100%, *no movement at all of the upper limb against gravity*; 66%, *flexion and/or extension movements in the hand but not in the arm*; 33%, *flexion and/or extension movements in both the hand and the arm against gravity but not against resistance*; 0%, *flexion and extension movements in both the hand and the arm against resistance*.

The block was considered to be complete if the dermatomes of the nerves implicated in the surgical site were anaesthetised. All nerves of the surgical site including those of the skin, muscles, and bones were considered. The block was evaluated as incomplete and in need of completion before surgery if one of the nerves of the surgical site was not anesthetized.

All data were collected in an Excel^®^-Sheet for documentation. For statistical analysis, the program SPSS 13.0 ^® ^for Windows (LEAD Technologies Inc, USA, 2004) was used. Differences in the onset times and anesthesia between the four nerves were tested using Friedman Repeated Measures Analysis of Variance (ANOVA) on Ranks. Statistical signicance was defined as p < 0.05.

## Results

29 female and 31 male patients were enrolled in the study. The demographic data and ASA status of the patient group are shown in Table 1. No difference among two groups were found with regard to the demographic data.

**Table 1:** Demographic data*

Group	Age (year)	Gender	Height (cm)	Weight (kg)	ASA Status
	(mean ± SD)	(M/F)	(mean ± SD)	(mean ± SD)	(ASA-1/ASA-2)
1 (US)	37.07 ± 16.24	13/17	167.01 ± 8.69	77.41 ± 14.85	14/16

2 (PNS)	39.96 ± 11.27	18/12	163.56 ± 7.24	74.49 ± 11.26	12/18

*No significant difference between the two groups.

The time needed to perform the AXB averaged is similar (resembling) in the two groups (p > 0,05) (Table 2). Time including sonographic overview and identification of the targeted structures (for Group 1), identification of the nerves via peripheral nerve stimulator (for Group 2), subcutaneous infiltration of the injection site, and application of local anesthetic (LA) to the direct vicinity of the four targeted nerves (in both Group 1 and 2).

**Table 2:** Block time and sensory block in 4 nerves.

	Group 1 (US) (n = 30)	Group 2 (PNS) (n = 30)	p value
	
Block time (min)	7.3 ± 2.6	6.4 ± 3.9	0.39
Sensory block in 4 nerves			

10 min	13 (43.33%)	9 (30.00%)	0.29

20 min	24 (80.00%)	17 (56.67%)	0.21

30 min	30 (100.00%)	26 (86.67%)	0.67

Although not significant statistically, it was observed that the sensory block had formed earlier in Group 1 (Table 2). The degree of motor blockade was intenser in group 1 than in Group 2 (p < 0.05) (Table 3). The succes rate of the sonographically guided axillary plexus block was 100%.

**Table 3:** Motor blockade.

Group	Motor blockade (%)			
	
	0% (n)	33% (n)	66% (n)*	100% (n)*
1 (US)	0	0	0	30

2 (PNS)	0	0	7	23

*significant difference between the two groups (66% and 100%)(p < 0.05).

There were neither cardiovascular side effects nor any accidental vascular punctures. There were no postoperative neurological symptom reported.

## Discussion

There are various techniques to block the brachial plexus clavicle at different levels from both under and above. Lately, most of the techniques used to inject the local anesthetics stipulate the use of paraesthesia. However, frequency of neurologic complications that occur following the AXB, varies between 0.2 and 19%. This may occur as a result of a direct trauma to the nerve, local anesthetic toxicity, ischaemia or a combination of all these factors [6,7].

The spread of LA around all nerves is obligatory to achieve complete AXB. Anatomical studies show the neurovascular space to be divided by multiple septae [8]. This is the main reason for incomplete AXB. Two different methods for solving the problem are used. One is the use of high LA volumes to achieve a good distribution in the axillary sheath [9]. This metod has a low risk of nerve damage so the cannula is not redirected in an area already anaesthesised. But incomplete blockades occur in patients with firm tissue surronding the nerves.

The more effective second metod is the multiple approach to terminal nerve branches by using nerve stimulation [10,11]. Nerve stimulators, that were first applied in 1912 however put into clinical application in 1962, have been an alternative to the technique of paraesthesia. It was believed that nerve stimulator minimized the possbility of a probable neuropathy that could be caused by a direct acute physical contact with the nerve with the paraesthesia technique. But this method increases the risk of nerve damage by redirecting the cannula in a previously anaesthesised area. Therefore, paraesthesia as a warning sign loses its value [12]. Fanelli at al^10^, reported a rate of 1.7% transient neurological complications using a multiple injection technique for peripheral nerve blockade.

The ultrasound approach identifies nerves, vessels, muscles, and septa. One main advantage of the sonographical approach is the ability to monitor the whole procedure of nerve blockade. Damage to important structures like vessels can be avoided during the puncture. We had no accidental vessel puncture in any patient too. Therfore, redirecting the cannula can be performed under visual control. The risk of accidental nerve damage can thus possibly be reduced. On the other hand, not only does ultrasonography give us the opportunity to observe the LA solution surrounding the nerve but also it lets us observe the optimal distribution of the injected LA solution around the nerve.

In our study, 86.67% of the cases in Group 2 (PNS) formed a sensory full block and 76.67% of these formed a motor full block within the first half hour.(Table 2 and 3). On the other hand, in Group 1 (US) sensory full block and motor full block rates were 100%. The fact that we receive better results following the US application is mainly caused by the possibility of observing the nerves forming the brachial plexus and the distribution of local anaesthetic liquid. Whether the consequently applied LA liquid had completely reached the targeted tissues or not can also be monitored.

Besides, ultrasonography can also be used for difficult axillary block applications [13]. Li et al [14] reported that ultrasonography is very useful in terms of application especially for obese cases.

Schwemmer et al [15,16] stated that ultrasonography application significantly increases the success rate of axillary blocks and that starting time of operation following the block is much earlier. Throughout our study, we detected that sensory block started earlier in the ultrasonography-applied group although this was not singnificant statistically and on the other hand, that motor block rate in this group was significantly higher in comparison with the other group.

Soeding et al [17] detected that ultrasonography application significantly reduced the starting time of sensory and motor block and that it significantly increased the block quality. Kefalianakis et al [18] stated that ultrasonography application decreases the starting of block. In our study, we have identified that sensory block start was earlier in the ultrasonography-applied group although that was not statistically significant.

According to Liu et al [19], ultrasonography application provides more accomplished sensory and motor blocks. Same researchers also reported that, through ultrasonography they managed to provide a highly sufficient analgesia without any complications in sixteen axillary-block applied cases of final-stage renal failures^20^. We did not encounter any serious complications in our ultrasonography-applied group throughout the study.

## Conclusions

Consequently, we detected that sensory block started earlier in the ultrasound-guided AXB although that was not statistically significant and that, however, success rate of motor block was higher. We believe that ultrasonography application especially, can be a good alternative without causing any compliations for cases with anatomic complexities.

## Consent

Written informed consent was obtained from the patient's for the publication of this case report and accompanying images. A copy of the written consent is available for review by the Editor-in-Chief of this journal.

## Competing interests

The author declares that they have no competing interests.

## Authors' contributions

BZ presented the cases history, performed cases management, drafted the manuscript; The author read and approved the final manuscript.
